# Novel Adsorbent Based on Banana Peel Waste for Removal of Heavy Metal Ions from Synthetic Solutions

**DOI:** 10.3390/ma14143946

**Published:** 2021-07-14

**Authors:** Mihai Negroiu, Anca Andreea Țurcanu, Ecaterina Matei, Maria Râpă, Cristina Ileana Covaliu, Andra Mihaela Predescu, Cristian Mircea Pantilimon, George Coman, Cristian Predescu

**Affiliations:** 1 Faculty of Biotechnical Systems Engineering, University Politehnica of Bucharest, 313 Spl. Independentei, 060042 Bucharest, Romania; negroiu.mihai@yahoo.com (M.N.); cristina_covaliu@yahoo.com (C.I.C.); 2 Center for Research and Eco-Metallurgical Expertise, University Politehnica of Bucharest, 313 Spl. Independentei, 060042 Bucharest, Romania; 3 Faculty of Materials Science and Engineering, University Politehnica of Bucharest, 313 Spl. Independetei, 060042 Bucharest, Romania; rapa_m2002@yahoo.com (M.R.); andra.predescu@upb.ro (A.M.P.); mircea.pantilimon@upb.ro (C.M.P.); george.coman@upb.ro (G.C.); cristian.predescu@upb.ro (C.P.)

**Keywords:** alginate, chitosan, biochar, eco-materials, heavy metals, adsorption

## Abstract

Due to its valuable compounds, food waste has been gaining attention in different applications, such as life quality and environment. Combined with circular economy requirements, a valorization method for waste, especially banana waste, was to convert them into adsorbents with advanced properties. The banana waste, after thermal treatment, was used with high removal performances (100%) for the removal of heavy metals, such as Cr, Cu, Pb, and Zn, but their small particle size makes them very hard to recover and reuse. For this reason, a biopolymeric matrix was used to incorporate the banana waste. The matrix was chosen for its remarkable properties, such as low cost, biodegradability, low carbon footprint, and reduced environmental impact. In this research, different types of materials (simple banana peel ash BPA and combined with biopolymeric matrix, ALG–BPA, CS–BPA) were prepared, characterized, and tested. The materials were characterized by means of attenuated total reflection Fourier transform infrared spectroscopy (ATR-FTIR), optical microscopy (OM), scanning electron microscopy (SEM), and tested for the removal of metal ions from synthetic solutions using atomic absorption spectroscopy (AAS). The ALG–BPA material proved to be the most efficient in the removal of heavy metal ions from synthetic solution, reaching even 100% metal removal for Cr, Fe, Pb, and Zn, while the CS-based materials were the least efficient, presenting the best values for Cr and Fe ions with a removal efficiency of 34.14% and 28.38%, respectively. By adding BPA to CS, the adsorption properties of the material were slightly improved, but also only for Cr and Fe ions, to 37.09% and 57.78%.

## 1. Introduction

Some of the biggest problems the world is facing today are gravitating around waste and pollution. Resources are extracted, processed, used, and ultimately stored as waste. At the end of the life cycle, the waste is usually incinerated (or heat processed) or stored in the field. Circular economy tries to combat both these problems by applying the “3 Rs”: Reduction (demand and/or consumption of resources, materials, and products), Reuse, and Recycling (return of materials to another life cycle) [1]. In this case, waste is always considered a value-added substance. The conventional methods to deal with food waste include thermal and chemical treatment that generate smaller molecules (CO, CO_2_, CH_4_, H_2_O, NH_3_, etc.), solids (compost, slag, and ash), but the environmental risk of air pollution does not disappear, contributing to the increase in greenhouse gases and the amount of waste and wastewater [2]. By comparison, the recovery of waste through emerging technologies does not lead to the destruction of nutrients or other useful components, and thus, they can be transformed into value-added products. In this way, waste of this type for which emerging recovery technologies are applied minimizes losses by becoming resources in the context of the circular economy.

One of the most crucial necessities of life is having access to clean, unpolluted waters. A lot of factors contribute to and cause water pollution, factors such as the increase in human population, urbanization, industrialization, agriculture activities, chemical use, etc. [3]. Some of the most persistent and non-biodegradable pollutants related to industrial discharges, agriculture, and mining are heavy metals [4]. Polluted waters contain heavy metals such as cadmium, arsenic, cobalt, chromium, copper, lead, and zinc [5]. These are dangerous if they exceed the permissible limit in the human body, causing chronic illness and even death [4]. Usually, for the removal of different pollutants from waters, materials such as activated carbon [6,7] and ion exchange resins [8,9,10,11] are used, but since these materials are expensive, there is a need for new, low cost, biodegradable eco-materials to use for removal of heavy metals from polluted waters.

Due to their biodegradability, biocompatibility, unique structure, low cost, and reduced environmental impact, biopolymers obtained from renewable resources such as polysaccharides (alginates, chitosan, starch, etc.) have gained considerable attention for materials used in the depollution of waters [12,13,14,15,16,17,18,19]. Besides these sources, another way to obtain low-cost materials is to use the waste from other sectors of industry, for example, different types of waste from the food industry. Food waste is classified into two major groups: animal (dairy, coal, fish, and seafood processing industry, etc.) and vegetable (from cereals, tubers, roots, to oil crops, fruits, and vegetables), and because of their perishable nature, combined with various external factors, it is difficult to capitalize on them. The common factor that is present in all these different types of waste is the high amount of organic matter, such as carbohydrates, proteins, lipids, and bioactive compounds [20].

These materials also present a high number of functional groups, such as the hydroxyl groups, which are responsible for the removal of heavy metal ions by biosorption [21]. Alginate has been studied by many researchers in the heavy metal removal from wastewaters. Lagoa et al. [22] discovered in their work that dried alginate beads are more adsorbent due to the higher surface area. Alginate functionalized with other materials, such as activated carbon [23], biochar from biomass [24,25], carbon nanotubes [26], graphene [15], and other biopolymers [27,28] have also proved to be efficient in the removal of metals such as Cd^2+^ [29,30], Cu^2+^ [23,31], Mg^2+^, Fe^2+^ [32], Pb^2+^ [23,31], and Zn^2+^ [31].

Chitosan and its derivatives have also been used in the heavy metal removal from contaminated waters. In the adsorption process, the metal ions bind to the amino groups and hydroxyl groups [21]. These kinds of materials have been tested for the removal of metal ions such as Cu(II) [33,34], Cd(II), Pb (II) [35], and Cr (VI) [36].

Among the multitude of agricultural waste tested as an adsorbent, banana waste has been of significant importance because there are parts that can be used, such as shells, trunks, pseudo-stem, leaves, and core. These parts of banana waste have been extensively studied as an adsorbent for wide ranges of pollutants [4]. Of the types of waste resulting from the consumption of fresh fruit, banana waste (mainly peel) is in first place, contributing 29% to the total waste generated in this category [5]. Ash obtained from different waste biomass has been also recognized as an adsorbent for metal removal from polluted waters. Ash obtained from wood-burning has proved to be efficient for the removal of Mn^2+^ ions from wastewater [37], Cr^4+^ ions [38]. Borlodoi et al. studied how ash obtained from banana peels can remove iron ions from groundwater from a concentration of 20 mg/L to 0.3 mg/L [39]. Das et al. [40] obtained ashes from bamboo, banana leaf, banana rind, banana pseudo-stem, and rice husk, tested them for the removal of Fe^2+^ ions from water samples and found the most efficient to be the banana pseudo-stem ash.

The objective of the present study was to prepare, characterize and test four new eco-materials based on banana peel ash, sodium alginate, and chitosan in aqueous solutions containing heavy metal ions as the first matrix for future experiments regarding real industrial waters.

## 2. Materials and Methods

### 2.1. Chemicals and Reagents

To prepare the eco-materials consisting of biopolymers (alginate-ALG, chitosan-CS) and banana peel ash (BPA) microbeads, the following reagents and precursors were used: Sodium alginate (BioChemica, Billingham, UK), (C_6_H_7_O_6_Na)_n_, MW = 10,000–600,000 g/mol, chitosan medium molecular mass was provided by Sigma-Aldrich, Darmstadt, Germany, banana peels to obtained the BPA, CaCl_2_, and glutaraldehyde as reticulation agents, sodium hydroxide (NaOH) pellets for analysis (max. 0.02% K) EMSURE^®^ ACS, Reag. Ph Eur, ISO (Merck, Darmstadt, Germany), acetic acid (glacial) 100% Suprapur^®^ (Merck, Darmstadt, Germany). For the removal efficiency testing, an ICP multi-element standard solution IV (23 elements in diluted nitric acid), 1000 mg/L: 1000 mg/L: Ag, Al, B, Ba, Bi, Ca, Cd, Co, Cr, Cu, Fe, Ga, In, K, Li, Mg, Mn, Na, Ni, Pb, Sr, Tl, and Zn Certipur^®^) (Merck, Darmstadt, Germany) was used.

### 2.2. Preparation of the Banana Peel Ash (BPA)

A modified method proposed by Yang et al. [41] was used to obtain BPB, where the banana peels were charred and then activated using a 30% NaOH solution instead of KOH. To obtain the BPB, banana peels were dried in an oven at 140 °C for 4 h. The resulting banana peels were then subjected to two-stage pyrolysis in a calcination furnace. In the first stage of pyrolysis at a temperature of 700 °C, for 1 h, and a heating rate of 3 °C/min, amorphous coal was obtained. To remove partially charred intermediate compounds, the product was treated with NaOH at a weight ratio of 1:3, followed by a heating process at 800 °C for 1 h. After these steps, the BPA was washed with distilled water and dried until constant mass.

### 2.3. Preparation of the Alginate Microbeads

**Figure 1:**
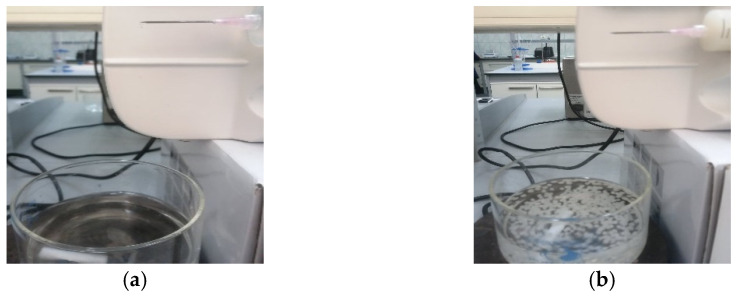
Dripping of 1% ALG solution (**a**) and ALG–BPA (1:1) (**b**) in a CaCl_2_ bath.

To prepare the ALG and ALG–BPA (1:1) microbeads, first, a 1% solution was prepared by dissolving the desired amount of sodium alginate in hot water and under vigorous stirring. The formed viscous solution was put in an ultrasound bath to eliminate the air bubbles. Half of the solution was put aside to later prepare the ALG microbeads, and the other half was mixed with the BPA to form the ALG–BPA (1:1) microbeads. The ALG and ALG–BPA blend were dripped in a 2% CaCl_2_ bath for reticulation and microbead formation, as can be seen in Figure 1.

**Figure 2:**
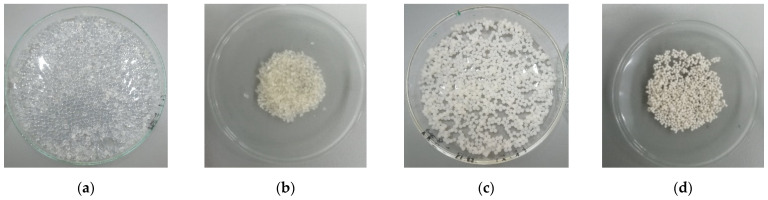
The appearance of ALG (wet—(**a**), dried—(**b**)) and ALG–BPA (1:1) (wet—(**c**), dried—(**d**)) microbeads.

The beads were left overnight for reticulation in the CaCl_2_ solution and then washed with distilled water until the total removal of Cl^−^ ions (testing with AgNO_3_). After washing, the beads were left to dry overnight, and they are presented in Figure 2.

### 2.4. Preparation of the Chitosan Microbeads

**Figure 3:**
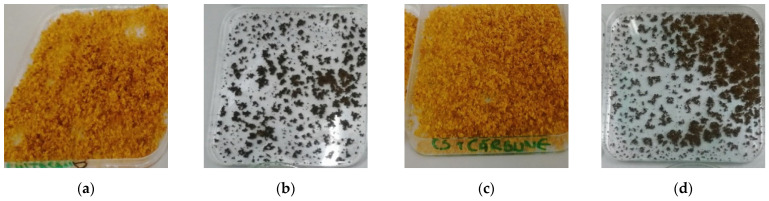
The appearance of CS (wet—(**a**), dried—(**b**)) and CS–BPA (1:1) (wet—(**c**), dried—(**d**)) microbeads.

To prepare the CS and CS–BPA (1:1) microbeads, first, a 1% solution was obtained by dissolving the desired amount of chitosan in 1% acetic acid and under vigorous stirring. The formed viscous solution was put in an ultrasound bath to eliminate the air bubbles. Half of the solution was put aside to later prepare the CS microbeads, and the other half was mixed with the BPA to form the CS–BPA (1:1) microbeads. The CS solution and the CS–BPA blend were crosslinked with glutaraldehyde for reticulation and the blends started to separate as the microbeads were forming. Then, the materials were washed abundantly with distilled water using a Büchner funnel. After washing, the materials were left to dry overnight and are presented in Figure 3.

### 2.5. Characterization of the Microbeads

Attenuated total reflectance Fourier transform infrared spectroscopy (ATR-FTIR) (Interspec 200-X Spectrophotometer, Interspectrum, Tõravere, Estonia) was used to characterize the samples before and after the addition of BPA.

To determine the structure and morphology of the eco-materials, Optical Microscopy (OM) (OLYMPUS BX51 M microscope, Tokyo, Japan) and Scanning electron microscopy (SEM) coupled with energy-dispersive spectra EDS (QUANTA 450 FEG microscope, Eindhoven, The Netherlands), equipped with a field emission gun and a 1.2 nm resolution X-ray energy dispersive spectrometer, with a resolution of 133 eV) were used.

### 2.6. Removal Efficiency Testing of the Materials

Batch adsorption experiments analysis of a multielement aqueous solution were performed by using a ContrAA^®^ 800D Atomic Absorption Spectrometry (AAS) system from Analytik, Jena, Germany, with acetylene flame with specific wavelengths between 185 and 900 nm. In total, 0.5 g of each material was put in contact with 100 mL of a multielement solution of 0.5 mg/L concentration. The water solutions containing the metal ions were prepared by diluting the standard solution with purified water. These solutions were in the pH range 4–5. The materials were left in contact with the solution for one hour, and every 15 min, samples were taken from the solution to test the remaining concentration of metal ions selected in the study (Cd, Co, Cr, Cu, Fe, Mn, Pb, and Zn). From the absorbance given by the AAS, the concentrations were calculated, and the removal efficiency was calculated using the following formula:

$$\mathrm{Removal}\;\mathrm{efficiency}\;\left(\%\right)=\frac{C_{i}-C_{f}}{C_{i}}*100$$

where $C_{i}$ was the concentration of the water solution before contact with adsorbante material (at 0 min), and $C_{f}$ was the concentration of the solution after a certain amount of time (*f* = 15, 30, 45, 60 min).

## 3. Results and Discussion

### 3.1. ATR-FTIR Analysis

#### 3.1.1. BPA

**Figure 4:**
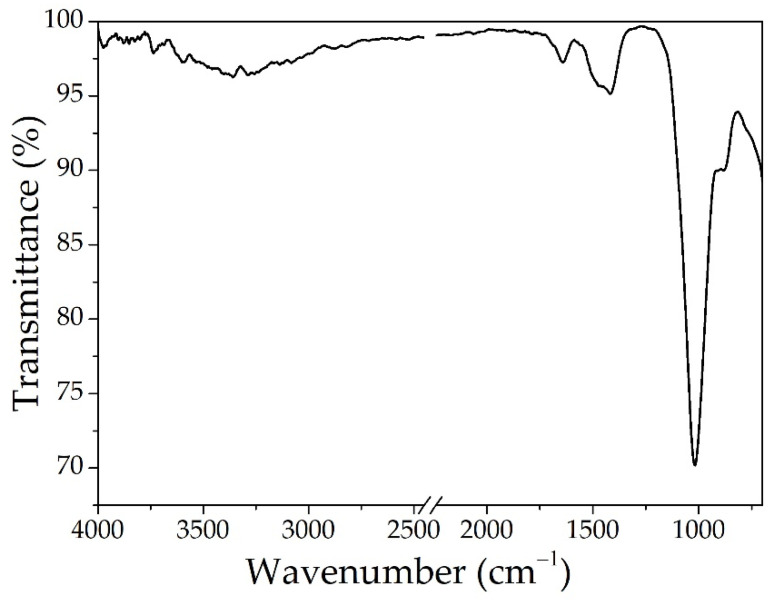
The ATR-FTIR analysis for BPA.

The spectrum for the BPA sample presented in Figure 4 shows a peak located at 3358 cm^−1^, which is attributed to the stretching vibration of the O-H bonds, that suggests that the material contains amorphous silicate or possible aluminum silicate and the peak at 1639 cm^−1^ is attributed to bending vibrations of H_2_O molecules [42]. The peaks at 1419 cm^−1^ and 1018 cm^−1^ are specific for stretching vibration of Si-O-Si and Al-O-Si bonds [43].

#### 3.1.2. ALG and ALG–BPA Microbeads

**Figure 5:**
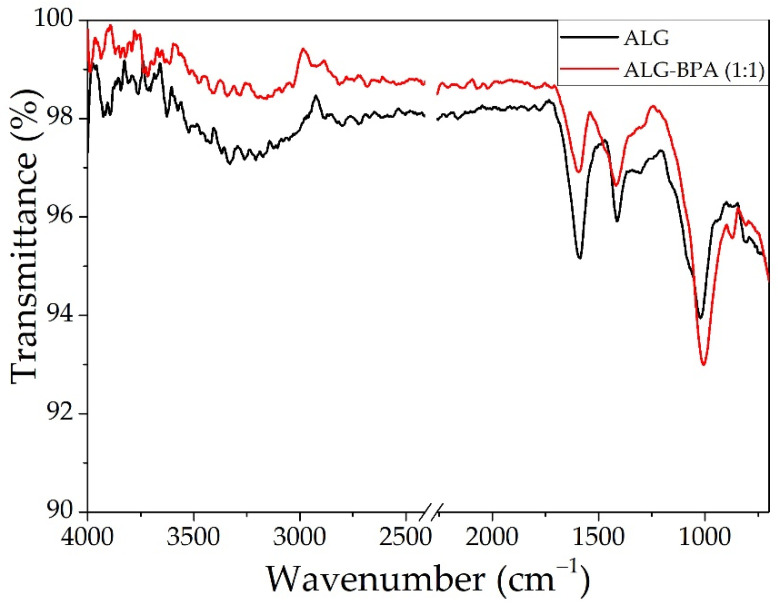
The ATR-FTIR analysis for ALG and ALG–BPA (1:1) microbeads.

The ATR-FTIR analyses for the alginate-based microbeads are presented in Figure 5, and the peak data are presented in Table 1. The spectrum for the ALG sample presents peaks from stretching and bending vibration given by the basic functional groups present in the calcium alginate chemical structure. Stretching vibrations of O–H bonds of alginate appear in the range of 3000–3600 cm^−1^, and the stretching vibrations of aliphatic C–H were observed at 2920–2850 cm^−1^. The bands at 1650 and 1400 cm^−1^ were attributed to asymmetric and symmetric vibrations of carboxylate salt ions and 1029 cm^−1^ (C–O stretch, primary hydroxyl group) [27,31,44]. The ALG–BPA spectrum has a lower intensity for the OH band, indicating the encapsulation of the BPA in the ALG.

#### 3.1.3. CS and CS–BPA Microbeads

**Figure 6:**
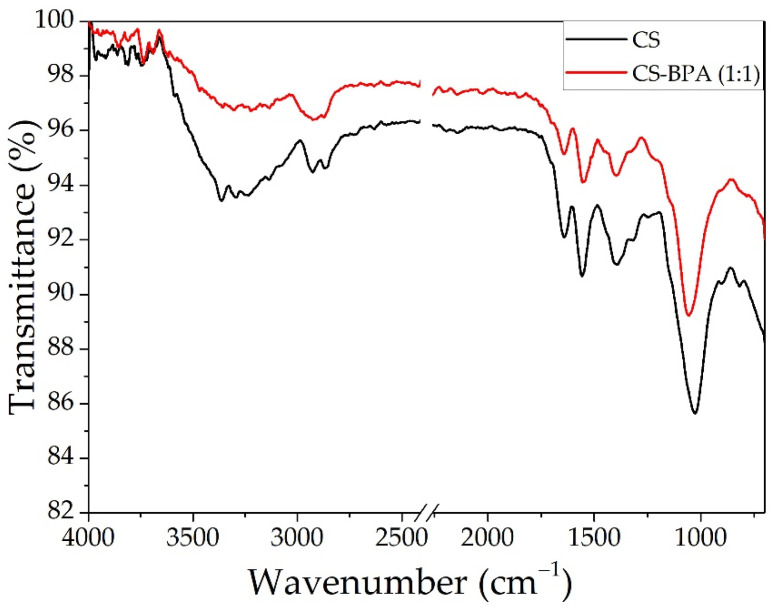
The ATR-FTIR analysis for CS and CS–BPA (1:1) microbeads.

The ATR-FTIR analyses for the chitosan-based microbeads are presented in Figure 6, and the peak data are presented in Table 2. The basic characteristic peaks of the chitosan were shown at 3308 cm^−1^ (O–H stretch and N–H stretch, overlapped), 2929 and 2865 cm^−1^ (C–H stretch), 1641 cm^−1^ (NH_2_ deformation), 1556 cm^−1^ (N–H bend), 1029 cm^−1^ (C–O stretch, primary hydroxyl group), as reported in the literature [27,45]. When compared to the CS spectrum, the one for CS–BPA shows that the peak for O–H stretch and N–H stretch has a lower intensity, meaning that the BPA is bounded to the CS chemical structure.

### 3.2. Optical Microscopy Analysis

#### 3.2.1. ALG and ALG–BPA Microbeads

**Figure 7:**
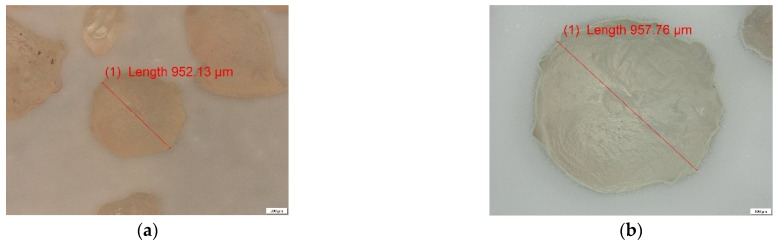
Optical microscopy analysis for ALG microbeads: (**a**) 50× and (**b**) 100× magnification.

**Figure 8:**
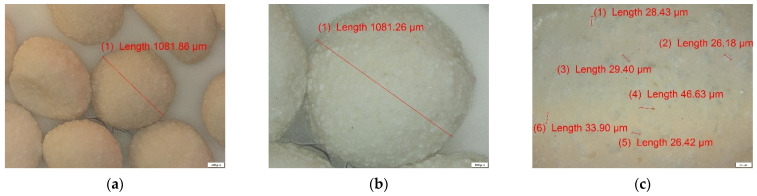
Optical microscopy analysis for ALG–BPA (1:1) microbeads: (**a**) 50×, (**b**) 100×, and (**c**) 200× magnification.

ALG and ALG–BPA (1:1) microbeads were analyzed by optical microscopy (OM) using a polarized light optical microscope. The images obtained at magnifications of 50, 100 and 200×, respectively, are shown in Figure 7 for the simple ALG sample and Figure 8 for the ALG–BPA 1:1 sample.

It can be observed that for the ALG sample, the size is located in the range of 900–1200 µm, these being characteristic of the particles after dehydration. They maintain their spherical shape, as can be seen in Figure 7. The incorporation of BPA in the ALG mass can be seen in Figure 8, the white particles of BPA being evenly distributed in the mass of ALG, having dimensions in the range of 20–50 µm.

#### 3.2.2. CS and CS–BPA (1:1) Micro Beads

**Figure 9:**
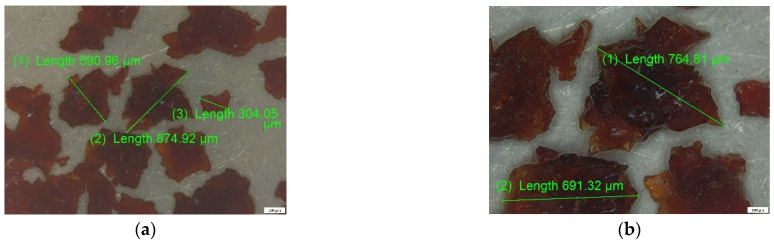
Optical microscopy analysis for CS microbeads: (**a**) 50× and (**b**) 100× magnification.

**Figure 10:**
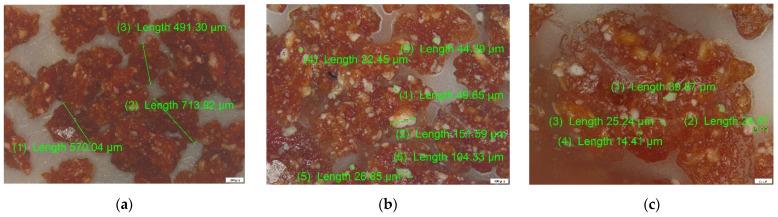
Optical microscopy analysis for CS–BPA (1:1) microbeads: (**a**) 50×, (**b**) 100×, and (**c**) 200× magnification.

CS and CS–BPA microbeads were analyzed by optical microscopy (OM) under a polarized light optical microscope. The images obtained at magnifications of 50, 100 and 200×, respectively, are shown in Figure 9 for the simple CS sample and Figure 10 for the CS–BPA 1:1 sample.

It can be observed in Figure 8 that the size of crosslinked CS particles is located in the range of 300–900 µm, but also the glassy aspect of the surface, these being characteristic of CS particles, after dehydration. The incorporation of BPA in the CS mass can be observed in Figure 9 by the white particles in the dehydrated CS mass, and respecting the same magnification order, the size of the BPA aggregates in the range 20–150 µm.

### 3.3. SEM-EDS Analysis

#### 3.3.1. BPA

**Figure 11:**
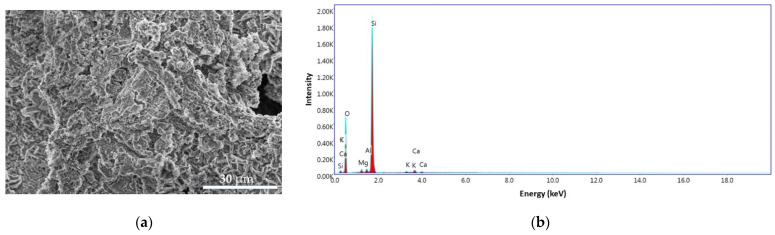
SEM images for BPA sample at 4000× (**a**) and the EDS pattern (**b**).

In order to study the morphology of the BPA surface obtained from banana peel waste, scanning electron microscopy (SEM) analyses, coupled with energy dispersive spectroscopy (EDS), were performed and also to identify the elements present in the sample. As can be seen in Figure 11a, the surfaces show homogeneity and porous appearance of the ash particles. The EDS pattern for the BPA sample (Figure 11b) exhibit a carbon content of 6.68%, oxygen 53.44%, magnesium 1.31%, aluminum 1.02%, silicon 35.97%, potassium 0.47%, and calcium 1.12%.

#### 3.3.2. ALG and ALG–BPA Microbeads

**Figure 12:**
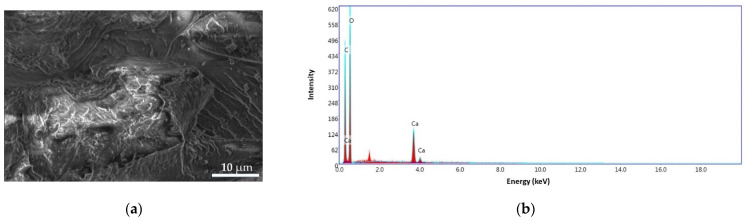
SEM images for ALG microbeads at 8000× (**a**) and the EDS pattern (**b**).

**Figure 13:**
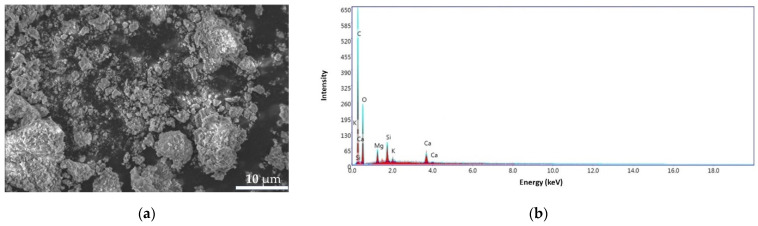
SEM images for ALG–BPA (1:1) microbeads at 8000× (**a**) and the EDS pattern (**b**).

The SEM-EDS analysis was used to determine the surface morphologies of ALG and ALG–BPA (1:1) microbeads, and it is presented in Figure 12 and Figure 13. For the ALG sample (Figure 12a), the surface revealed nonaggregate microspheres with irregular surfaces, high roughness, and cracks caused by collapsing of the polymer layers during dehydration. Similar results were reported by Lagoa et al. [22]. The energy dispersive X-ray (EDS) patterns of ALG (Figure 12b) exhibit carbon content of 38.84 wt.%, oxygen of 58.34 wt.%, and the calcium content of 2.82 wt.%.

For the ALG–BPA (1:1) sample (Figure 13a), the surface shows small beads deposited in the cracks and all over the surface, indicating the encapsulation of the BPA in the ALG matrix. The energy dispersive X-ray (EDS) patterns of ALG–BPA (1:1) (Figure 13b) exhibit carbon content of 60.38 wt.%, oxygen 35.55 wt.%, calcium 1.03 wt.%, magnesium 1.46 wt.%, silicon 1.31 wt.%, and phosphorus of 0.27 wt.%. The presence of Si, Mg, and K elements is from adding the BPA in the ALG matrix.

#### 3.3.3. CS and CS–BPA Microbeads

**Figure 14:**
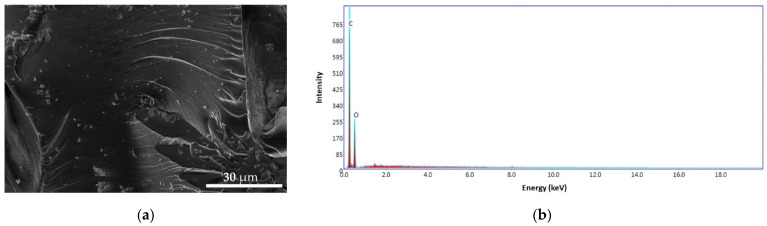
SEM images for CS microbeads at 4000× (**a**), EDS pattern (**b**).

**Figure 15:**
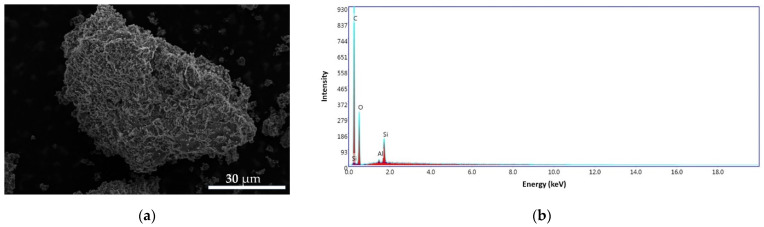
SEM images for CS–BPA (1:1) microbeads at 4000× (**a**) and the EDS pattern (**b**).

The morphology and structure of the CS and CS–BPA microbeads are presented in Figure 14 and Figure 15. The CS sample presents a nonporous, smooth membranous phase consisting of dome-shaped orifices, microfibrils, and crystallites. The energy dispersive X-ray (EDS) patterns of CS microbeads (Figure 14b) exhibit carbon content of 64.55 wt.% and oxygen of 35.45 wt.%.

In Figure 15a, we can see that the surface of the CS–BPA (1:1) sample is irregular, with different aggregates formed on the surface, indicating the encapsulation of BPA in the chitosan chemical structure. The energy dispersive X-ray (EDS) patterns of the sample (Figure 15b) exhibit carbon content of 64.74 wt.%, oxygen of 33.28 wt.%, silicon of 1.73 wt.%, and traces of aluminum. The Al appears due to the support on which the sample is analyzed in the SEM equipment.

### 3.4. Batch Adsorption Tests

#### 3.4.1. Testing of the BPA

**Figure 16:**
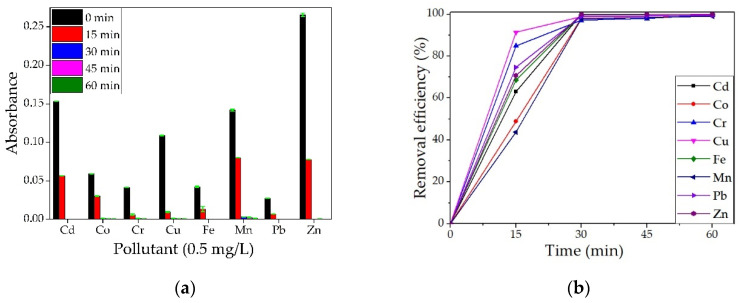
Qualitative analysis on a multielement solution (concentration 0.5 mg/L) for BPA (**a**) and removal efficiency (**b**).

Samples were taken for analysis every 15 min, up to 60 min, with three repetitions per experiment. The averages of the three absorbance values read for each element are represented graphically in Figure 16a.

It can be observed from Figure 16b that the removal efficiency is high for all the metals studied, reaching 100% for Zn after only 30 min of contact and for Cd and Fe after 60 min of contact. For all metals studied, the removal efficiency was over 97% after 30 min of contact. Similar results for different metal ions were obtained by other researchers when using wood ash. In a study by Mosoarca et al. [37], ash obtained from wood-burning was used to retain Mn^2+^ ions from wastewater. They found that the material presents an adsorption efficiency of over 90% but can be increased to over 98% by parameter control, with best values obtained for pH~6, a material dose of 10 g/L. Wood ash was also studied by Pehlivan et al. [38] for the removal of Cr^4+^ ions. They observed that the adsorption of Cr^4+^ ions was higher at a pH between 2 and 2.5. Borlodoi et al. studied how ash obtained from banana peels can remove iron ions from groundwater from a concentration of 20 mg/L to 0.3 mg/L [39]. Das et al. [40] obtained ashes from bamboo, banana leaf, banana rind, banana pseudo-stem, and rice husk, tested them for the removal of Fe^2+^ ions from water samples and found the most efficient to be the banana pseudo-stem ash.

Due to the small particle size that provides a high surface area and thus a maximum efficiency in removing metals, the regeneration process is difficult. Thus, after testing in multielement solution, for 60 min, the BPA material was dried and weighed, and it was observed that the recovery yield of the BPA material is about 25%; the losses thus influence the high efficiency of the material. Based on these findings, the possibility of incorporating BPA material in biopolymeric ecological matrices, such as chitosan and alginate, was studied.

#### 3.4.2. Testing the Polysaccharide-BPA Materials

**Figure 17:**
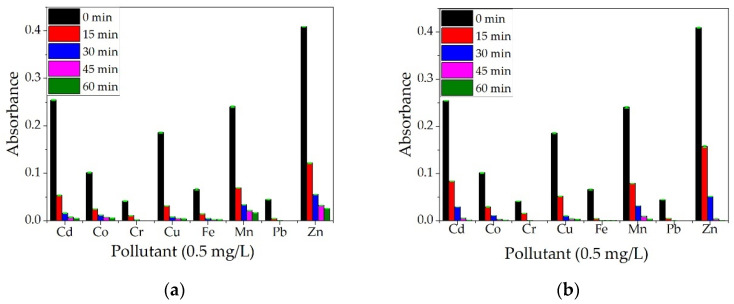
Qualitative analysis on a multielement solution (concentration 0.5 mg/L) for ALG (**a**) and ALG–BPA (1:1) microbeads (**b**).

The materials were agitated with a magnetic stirrer to enhance the metal adsorption, and samples were taken every 15 min to observe the decrease in metal ions concentration. The metals studied were Cd, Co, Cr, Cu, Fe, Mn, Pb, and Zn. It can be observed from Figure 17a,b, that, when using ALG and ALG–BPA materials, in a period of 1 h, the absorbance for most metal ions decreases significantly, indicating a decrease in metal ion concentration in the water sample. Similar results for ALG-based materials were observed by researchers. In a study by Rapa et al. [33], alginate was combined in different proportions with starch and nano clays to form some new composites for the removal of Cu^2+^ ions from waters. They observed that the optimal ratio between components alginate/starch/n-clay was 1/2/3 (dried components) with a maximum removal efficiency of 95% for Cu^2+^ ions after 25 h of contact. The removal of Cu^2+^, Mg^2+^, Fe^2+^, and Pb^2+^ by using alginate combined with different nanocellulose biosorbents was studied by Abou-Zeid et al. [32]. They found that tri-carboxylate cellulose nanofibers (TPC-CNF) combined with alginate presented high removal efficiency for the metal ions studied with the best values obtained for Pb (95%) and Cu (92%), but a lower removal efficiency for Mg (54%) and Fe (43%).

**Figure 18:**
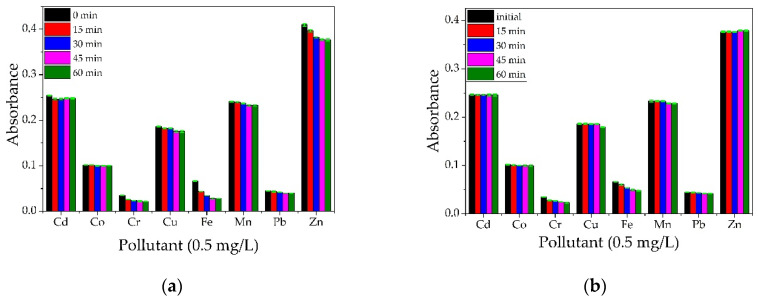
Qualitative analysis on a multielement solution (concentration 0.5 mg/L) for CS (**a**) and CS–BPA (1:1) microbeads (**b**).

For the CS and CS–BPA (1:1) microbeads, it can be observed in Figure 18a,b that the impact over the removal efficiency of heavy metal ions is less noticed in the case of the CS sample, while the CS–BPA sample proved to be efficient in partial removal of some metal ions. Ngah and Fatinathan [34] studied the removal of Cu(II) ions from an aqueous solution of chitosan beads, chitosan–GLA 1:1, 2:1 ratio beads, and chitosan–alginate beads, and based on their results, all materials are efficient for Cu(II) removal from water solutions. Modified chitosan was studied by Zhao et al. for the removal of Cd(II) and Pb (II) ions [35]. In a study by Matei et al. [36], magnetite nanoparticles were incorporated in a chitosan matrix and tested for the removal of Cr^6+^ ions from aqueous solutions. The biocompatible composite proved to be suitable for monolayer adsorption of Cr^6+^ ions on the porous surface of the material and presented a removal efficiency of 91% for a concentration of 0.5 mg/L Cr^6+^ ions.

**Figure 19:**
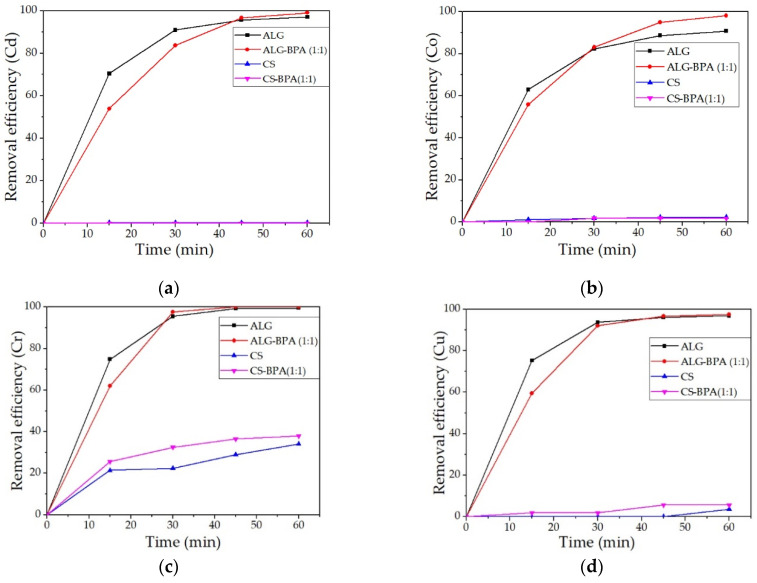

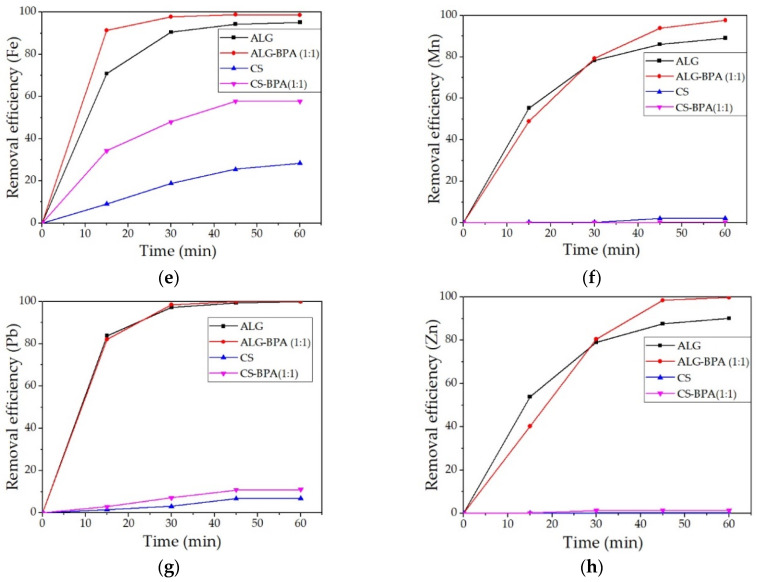
Removal efficiencies of the 4 types of microbeads for the metals studied: Cd (**a**), Co (**b**), Cr (**c**), Cu (**d**), Fe (**e**), Mn (**f**), Pb (**g**), and Zn (**h**).

The removal efficiencies for heavy metal ions from water solution for the four types of microbeads are presented in Figure 19a–h. It can be observed that the most efficient material for the removal of most metals studied is the ALG–BPA (1:1) microbeads. They present over 95% removal efficiency for the metals studied and 100% removal efficiency for Cr, Fe, Pb, and Zn. The ALG microbeads also presented good removal efficiencies of metal ions, with values over 90%. The least efficient material was the CS microbeads. The best values were obtained for Cr and Fe ions with a removal efficiency of 34.14% and 28.38%, respectively. By adding BPA to CS, the adsorption properties of the material were slightly improved, but also only for Cr and Fe ions, to 37.09% and 57.78%.

## 4. Conclusions

Banana waste was thermally treated to prepare a new eco-material with a high removal efficiency of heavy metal ions from synthetic solutions. The BPA obtained, although very efficient (over 97% removal efficiency for all metals investigated), presented a big problem with recovery and reuse, so the BPA was incorporated in biopolymeric matrixes (ALG and CS). These materials were tested in aqueous solutions containing heavy metal ions as the first matrix for future experiments regarding real industrial waters. The materials were characterized in terms of structural and morphological properties using ATR-FTIR, OM, SEM-EDS, and batch adsorption tests using AAS. From FTIR analysis, it was observed a lowering in the concentration of hydroxyl groups, indicating the presence of BPA in the structure of ALG and CS-based microbeads. From the OM and SEM-EDS analysis, the morphology and surface structure of the materials were studied, and it was observed that the materials with BPA presented an irregular surface and irregular aggregates. The most relevant results were obtained for ALG–BPA microbeads, which presented 100% removal efficiency for Cr, Fe, Pb, and Zn and above 90% for the other metal ions. The least adsorbent material was the one with CS, presenting results for Cr and Fe ions with a removal efficiency of 34.14% and 28.38%, respectively. By adding BPA to CS, the adsorption properties of the material were slightly improved but also only for Cr and Fe ions to 37.09% and 57.78%. Although adding BPA to CS improved the adsorption properties of the material, it was still less efficient than the ALG-based microbeads in the removal of heavy metals from water solutions.

## Figures and Tables

**Table 1:** Peak data for ALG and ALG–BPA (1:1) microbeads.

ALG Microbeads		ALG–BPA (1:1) Microbeads	
Peak At	Assigned	Peak At	Assigned
814	CH bending	871	CH bending
1022	C–O–C	1007	C–O–C
1413	carboxylate salt ion	1418	carboxylate salt ion
1592		1595	
2891	–CH	2910	–CH
3329	–OH	3342	–OH

**Table 2:** Peak data for CS and CS–BPA (1:1) microbeads.

CS Microbeads		CS–BPA (1:1) Microbeads	
Peak At	Assigned	Peak At	Assigned
817	CH bending	1057	C–O–C
898	CH bending	1315	C–N
1029	C–O–C	1396	CH_3_ bending
1315	C–N	1550	NH_2_
1391	CH_3_ bending	1640	C=O
1556	NH_2_	2921	C–H
1641	C=O	3308	–OH, NH_2_
2865	CH stretching	–	–
2929		–	–
3357	OH, NH_2_	–	–

## Data Availability

Not applicable.
